# Supplementary material for: Reaction-conditioned generative model for catalyst design and optimization with CatDRX
Source: Commun Chem. 2025 Oct 23;8:314. doi: 10.1038/s42004-025-01732-7 (PMC12550025; doi:10.1038/s42004-025-01732-7)
Supplement: Supplementary file 2 — Supplementary Information [file 42004_2025_1732_MOESM2_ESM.pdf]

Supplementary Information
Reaction-conditioned generative model for catalyst design and optimization with CatDRX

Apakorn Kengkanna[1], Yuta Kikuchi[1], Takashi Niwa[2], Masahito Ohue[1*]

[1]Department of Computer Science, Institute of Science Tokyo, Kanagawa, 226-8501, Japan.
[2]Graduate School of Pharmaceutical Sciences, Kyushu University, Fukuoka, 812-8582, Japan.

*Corresponding author(s). E-mail(s): ohue@comp.isct.ac.jp;
Contributing authors: kengkanna@li.comp.isct.ac.jp; kikuchi@comp.isct.ac.jp; niwa@phar.kyushu-u.ac.jp;

Supplementary Note 1 Reaction Datasets

This section provides an overview of the datasets and related statistics. Complete details, including the number of unique components in the reaction conditions and the dataset split sizes, are shown in Table S1.

Table S1: Full details of reaction datasets and statistics

Dataset	Target	Size	Reaction						Splitting	Splitting		
			Condition	(Reactant	Reagent	Product	Time)	Catalyst		Train	Val	Test
Open Reaction Database (ORD)	%Yield	52448	42968	40609	3021	39485	362	2304	Random (90/5/5)	47203	2622	2623
Buchwald-Hartwig (BH)	%Yield	3955	990	15	66	5	1	4	Random (Original)	2491	276	1188
		3955	990	15	66	5	1	4	Reagent (Test1)	2752	305	898
Suzuki-Miyaura (SM)	%Yield	5760	480	15	32	1	1	12	Random (Original)	3629	403	1728
		5760	480	15	32	1	1	12	Catalyst (Test1)	3888	432	1440
Ruthenium catalyzed	%Yield (All)	382	284	111	26	107	36	84	Random (70/10/20)	267	38	77
hydrogenation (RU)	%Yield (>50%)	294	241	101	19	98	36	49	Random (70/10/20)	205	30	59
Ullmann couplings (UM)	%Yield	1131	26	6	13	6	1	70	Random (70/10/20)	791	113	227
Lewis acid-mediated Suzuki-Miyaura (L-SM)	%Yield	29	1	1	1	1	1	29	Random (80/20)	23	-	6
Asymmetric hydrogenation (AH)	%ee	362	241	188	12	186	13	66	Random (70/10/20)	253	36	73
C-C cross-coupling (CC)	Energy	7054	1	1	1	1	1	7054	Random (80/10/10)	5643	705	706
Asymmetric Pictet–Spengler (PS)	$\Delta\Delta G^{\ddagger}$	804	331	239	37	239	1	153	Random (80/10/10)	643	80	81

Supplementary Note 2 Data Representation

This section describes the structures and features of data representations used in this study. The structure of catalyst matrix is displayed in Fig. S1. The one-hot encoding features in catalyst matrix is displayed in Table S2. The node and edge features of molecular graph for reactants, reagents, and product are summarized in Table S3.

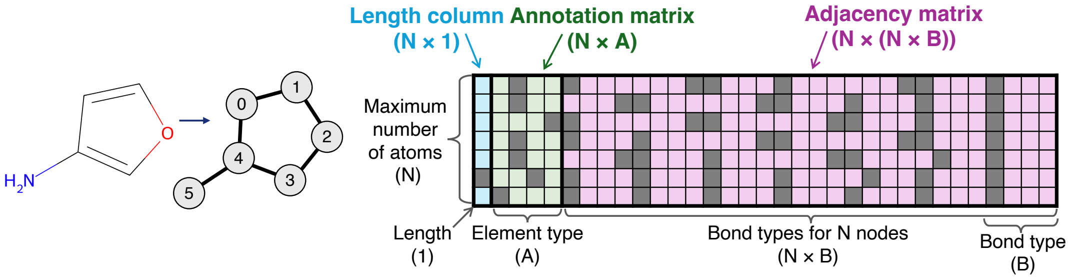

Fig. S1: Simplified catalyst matrix representation. This example utilizes maximum number of nodes at seven with four different atom types and four different bond types.

Table S2: Molecular matrix features

Matrix	Index	Description
Length column matrix (100)	0-99	Molecule size in one-hot
Annotation matrix (68×100)	0	No atom
	1-67	Atom's symbol (from pre-training dataset)
Adjacency matrix (6×100×100)	0	No bond
	1-5	Bond's type (single, double, triple, aromatic, dative)

Table S3: Molecular graph features

Feature	Index	Description
Node (101)	0-67	Atom's symbol (from pre-training dataset)
	68-78	Atom's degree (0-10)
	79-85	Atom's implicit valence (0-6)
	86	Atom's absolute formal charge
	87-89	Atom's formal charge sign (-, 0, +)
	90	Atom's number of radical electron
	91-95	Atom's hybridization (sp, sp2, sp3, sp3d, sp3d2)
	96	Atom's aromatic property
	97-98	Atom's chiral code (_CIPCode) (R, S)
	99	Atom's possible stereocenters (_ChiralityPossible)
	100	Atom's in ring property
Edge (11)	0-4	Bond's type (single, double, triple, aromatic, dative)
	5	Bond's conjugated property
	6	Bond's in ring property
	7-10	Bond's stereo type (STEREONONE, STEREOANY, STEREOZ, STEREOE)

Supplementary Note 3 Model Architecture Details

This section presents the details of model architecture including catalyst embedding architecture (Fig. S2) and decoder architecture (Fig. S3).

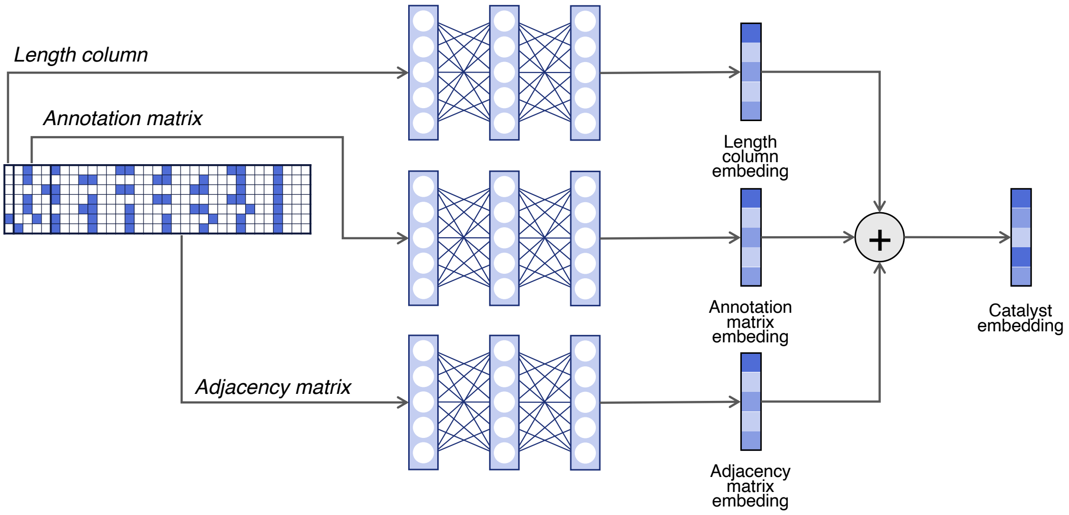

Fig. S2: Catalyst embedding architecture

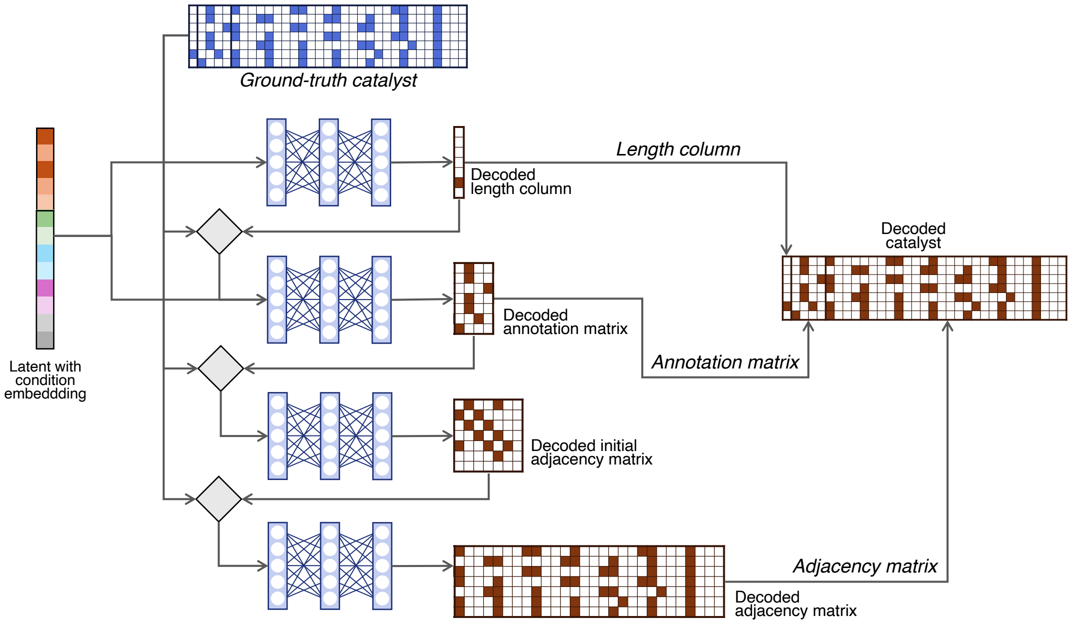

Fig. S3: Decoder architecture. The diamond shape represents random switching for teacher forcing to enhance decoder training. If teacher forcing is applied, the corresponding part of the ground-truth catalyst representation replaces the decoded structure before being supplied into the decoder networks.

Supplementary Note 4 Model Training Details

Key configuration and hyperparameters for pre-training and fine-tuning step are listed as follows: **Pre-training**. Configuration – catalyst embedding module: multiple linear layers, condition embedding module for molecular component: graph attention networks (GAT) with 3 layers and mean read-out function, predictor module: multiple linear layers, catalyst embedding dimension: 256, condition embedding dimension for each molecular component: 256, total condition embedding dimension: 776, latent embedding dimension: 256, reconstruction loss: cross-entropy, α parameter: 1, β parameter: 0.0001, KL-loss annealing: cosine shape with 100 steps, class weight: disabled, prediction loss: L1-loss, batch size: 256, epoch: 1000, learning rate: 0.0001, weight decay: 0.0005, optimizer: Adam, dropout: 0.1, augmentation: 5. A small beta was selected to encourage accurate reconstruction, given the diversity of catalyst structures, ranging from few atoms to larger multi-fragment systems. A smaller beta would help capture complex structural patterns and provide greater flexibility in the latent space. **Fine-tuning**. Hyperparameter – α parameter, β parameter, prediction loss, batch size, epoch, learning rate, learning rate scheduling, dropout.

Supplementary Note 5 Model Performance

This section presents the model prediction performance from benchmarking experiments and ablation studies. Prediction performance in terms of RMSE or MAE, compared to various benchmarks, is summarized in Table S4. Performance in terms of R^2 is shown in Fig. S4 and Table S5.

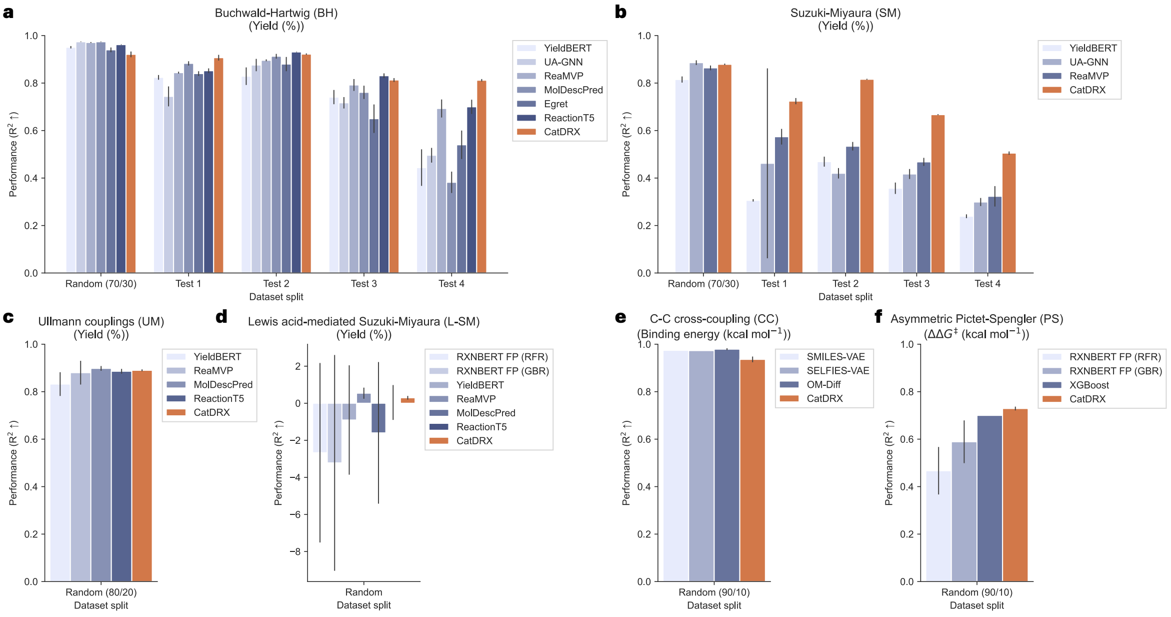

Fig. S4: Model prediction performance of several datasets in R^2

Results from the ablation studies are presented in Fig. S5 and Table S6. The results contain completed version of CatDRX with fine-tuning on pre-trained model with augmentation (FullCatDRX) and four alternative variants including pre-trained model without augmentation (NoAug), pre-trained model with augmentation but without fine-tuning (zero-shot with augmentation, NoFT), model without pre-training and augmentation (training directly on datasets, NoAug+NoPT), and pre-trained model without fine-tuning and augmentation (zero-shot without augmentation, NoAug+NoFT).

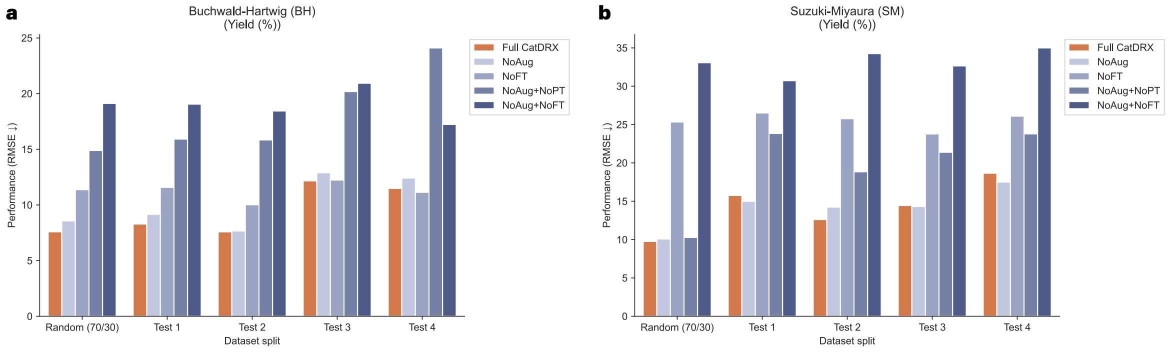

Fig. S5: Model prediction performance of ablation studies in RMSE.

Additional results comparing models trained using a pre-trained model and those trained on dataset only are visualized in Fig. S6. Models trained using a pre-trained model refers to models that were fine-tuned on the downstream task using previously pre-trained model. In contrast, models trained on the dataset only refers to models trained from scratch using only the downstream dataset.

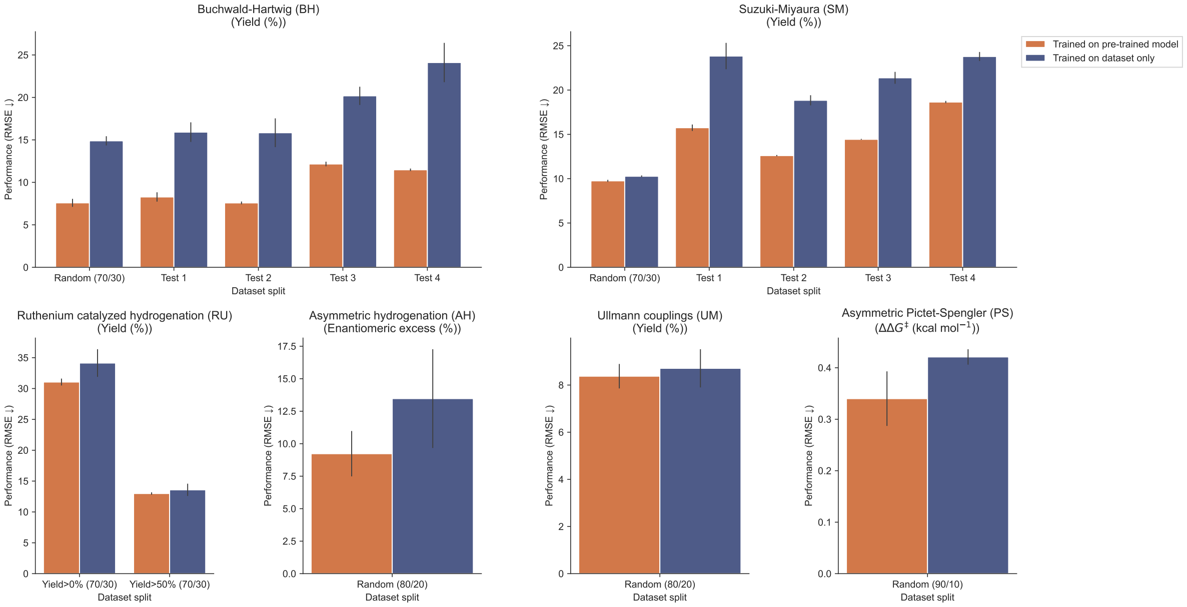

Fig. S6: Model prediction performance of ablation studies in RMSE comparing models trained using pre-trained model and models trained on dataset only

Table S4: Model prediction performance in RMSE or MAE

Dataset	Split	Metrics	Model							
			YieldBERT	UA-GNN	ReaMVP	MolDescPred	ReactionT5	CatDRX		
Buchwald-Hartwig (BH)	Random	RMSE ↓	6.014 ± 0.272	4.433 ± 0.085	4.626 ± 0.139	4.407 ± 0.089	5.265 ± 0.130	7.587 ± 0.472		
	Test 1	RMSE ↓	11.441 ± 0.342	13.746 ± 1.175	10.768 ± 0.136	9.320 ± 0.376	10.489 ± 0.270	8.282 ± 0.549		
	Test 2	RMSE ↓	11.144 ± 1.267	9.476 ± 1.027	8.722 ± 0.179	8.002 ± 0.472	7.094 ± 0.160	7.581 ± 0.163		
	Test 3	RMSE ↓	14.276 ± 0.820	14.939 ± 0.622	12.791 ± 0.769	13.726 ± 0.814	11.559 ± 0.290	12.162 ± 0.271		
	Test 4	RMSE ↓	19.679 ± 1.397	18.774 ± 0.566	14.618 ± 0.932	20.780 ± 0.767	14.465 ± 0.750	11.480 ± 0.146		
			YieldBERT	UA-GNN	ReaMVP	ReactionT5	CatDRX			
Suzuki-Miyaura (SM)	Random	RMSE ↓	12.073 ± 0.463	9.467 ± 0.459	10.367 ± 0.423	11.225 ± 0.390	9.755 ± 0.116			
	Test 1	RMSE ↓	25.000 ± 0.095	21.996 ± 0.818	19.564 ± 0.742	22.450 ± 0.350	15.750 ± 0.362			
	Test 2	RMSE ↓	19.592 ± 0.386	20.485 ± 0.391	18.357 ± 0.349	25.237 ± 0.020	12.606 ± 0.067			
	Test 3	RMSE ↓	20.051 ± 0.371	19.090 ± 0.342	18.236 ± 0.294	24.836 ± 0.660	14.440 ± 0.046			
	Test 4	RMSE ↓	23.114 ± 0.119	22.176 ± 0.270	21.796 ± 0.700	25.466 ± 0.510	18.647 ± 0.126			
			GP	RF	KNN	NN	YieldBERT	ReaMVP	MolDescPred	CatDRX
Ruthenium catalyzed hydrogenation (RU)	Yield>0%	RMSE ↓	25.460 ± 1.660	23.020 ± 0.500	30.020 ± 3.500	24.500	31.458 ± 2.700	31.108 ± 2.300	30.276 ± 1.180	31.052 ± 0.559
	Yield>50%	RMSE ↓	11.760 ± 0.840	13.600 ± 0.010	12.040 ± 0.950	14.100	13.888 ± 0.630	12.037 ± 1.600	12.695 ± 1.040	12.986 ± 0.195
			YieldBERT	ReaMVP	MolDescPred	ReactionT5	CatDRX			
Ullmann couplings (UM)	Random	RMSE ↓	9.974 ± 1.110	8.546 ± 1.570	8.398 ± 0.330	9.116 ± 0.970	8.376 ± 0.519			
			RXNBERT FP (RFR)	RXNBERT FP (GBR)	YieldBERT	ReaMVP	MolDescPred	ReactionT5	CatDRX	
Lewis acid-mediated Suzuki-Miyaura (L-SM)	Random	RMSE ↓	30.480 ± 6.703	30.942 ± 5.232	16.548 ± 5.940	16.288 ± 13.460	21.546 ± 8.520	18.372 ± 14.850	22.175 ± 13.880	
			RF	DT	GB	CNN	CatDRX			
Asymmetric hydrogenation (AH)	Random	RMSE ↓	8.406 ± 1.822	9.226 ± 1.888	9.620 ± 1.875	11.667 ± 2.831	9.231 ± 1.744			
			SMILES-VAE	SELFIES-VAE	BoB	Morgan	OM-Diff	CatDRX		
C-C cross-coupling (CC)	Random	MAE ↓	2.430	2.420	2.610	2.870	2.040 ± 0.080	4.020 ± 0.140		
			RXNBERT FP (RFR)	RXNBERT FP (GBR)	XGBoost	CatDRX				
Asymmetric Pictet-Spengler (PS)	Random	MAE ↓	0.411 ± 0.070	0.351 ± 0.070	0.256	0.250 ± 0.032				

Table S5: Model prediction performance in R^2

Dataset	Split	Metrics	Model						
			YieldBERT	UA-GNN	ReaMVP	MolDescPred	Egret	ReactionT5	CatDRX
Buchwald-Hartwig (BH)	Random	R2 ↑	0.951 ± 0.005	0.974 ± 0.001	0.971 ± 0.002	0.974 ± 0.001	0.940 ± 0.010	0.962 ± 0.000	0.921 ± 0.012
	Test 1	R2 ↑	0.824 ± 0.010	0.744 ± 0.042	0.844 ± 0.004	0.883 ± 0.009	0.840 ± 0.010	0.852 ± 0.010	0.907 ± 0.012
	Test 2	R2 ↑	0.829 ± 0.037	0.876 ± 0.026	0.896 ± 0.004	0.913 ± 0.010	0.880 ± 0.030	0.931 ± 0.000	0.922 ± 0.003
	Test 3	R2 ↑	0.741 ± 0.030	0.717 ± 0.024	0.792 ± 0.025	0.761 ± 0.028	0.650 ± 0.060	0.831 ± 0.010	0.813 ± 0.008
	Test 4	R2 ↑	0.444 ± 0.077	0.496 ± 0.031	0.693 ± 0.038	0.382 ± 0.045	0.540 ± 0.060	0.700 ± 0.030	0.812 ± 0.005
			YieldBERT	UA-GNN	ReaMVP	CatDRX			
Suzuki-Miyaura (SM)	Random	R2 ↑	0.815 ± 0.013	0.886 ± 0.010	0.864 ± 0.010	0.879 ± 0.003			
	Test 1	R2 ↑	0.306 ± 0.005	0.462 ± 0.400	0.574 ± 0.033	0.724 ± 0.013			
	Test 2	R2 ↑	0.469 ± 0.021	0.420 ± 0.022	0.534 ± 0.018	0.816 ± 0.002			
	Test 3	R2 ↑	0.357 ± 0.024	0.417 ± 0.021	0.468 ± 0.017	0.667 ± 0.002			
	Test 4	R2 ↑	0.239 ± 0.008	0.299 ± 0.017	0.323 ± 0.043	0.505 ± 0.007			
			YieldBERT	ReaMVP	MolDescPred	ReactionT5	CatDRX		
Ullmann couplings (UM)	Random	R2 ↑	0.832 ± 0.050	0.880 ± 0.050	0.898 ± 0.010	0.886 ± 0.010	0.890 ± 0.004		
			RXNBERT FP (RFR)	RXNBERT FP (GBR)	YieldBERT	ReaMVP	MolDescPred	ReactionT5	CatDRX
Lewis acid-mediated Suzuki-Miyaura (L-SM)	Random	R2 ↑	-2.671 ± 4.841	-3.218 ± 5.821	-0.904 ± 2.950	0.541 ± 0.300	-1.598 ± 3.820	0.041 ± 0.940	0.294 ± 0.100
			SMILES-VAE	SELFIES-VAE	OM-Diff	CatDRX			
C-C cross-coupling (CC)	Random	R2 ↑	0.974	0.973	0.979 ± 0.004	0.936 ± 0.012			
			RXNBERT FP (RFR)	RXNBERT FP (GBR)	XGBoost	CatDRX			
Asymmetric Pictet-Spengler (PS)	Random	R2 ↑	0.467 ± 0.100	0.589 ± 0.090	0.700	0.729 ± 0.008			

Table S6: Model prediction performance of ablation studies

Dataset	Splitting type	Splitting	Metrics	CatDRX	NoAug	NoFT	NoAug+NoPT	NoAug+NoFT
Buchwald-	Random split	Random (70/30)	R^2	0.921 ± 0.012	0.900 ± 0.017	0.824 ± 0.017	0.698 ± 0.026	0.503 ± 0.028
Hartwig (BH)	Out-Of-Sample	Test 1	R^2	0.907 ± 0.012	0.887 ± 0.006	0.820 ± 0.000	0.658 ± 0.048	0.512 ± 0.000
	Split (Reagent)	Test 2	R^2	0.922 ± 0.003	0.920 ± 0.002	0.863 ± 0.000	0.656 ± 0.072	0.537 ± 0.000
		Test 3	R^2	0.813 ± 0.008	0.789 ± 0.010	0.810 ± 0.000	0.483 ± 0.056	0.445 ± 0.000
		Test 4	R^2	0.812 ± 0.005	0.780 ± 0.010	0.823 ± 0.000	0.164 ± 0.156	0.576 ± 0.000
Suzuki-	Random split	Random (70/30)	R^2	0.879 ± 0.003	0.871 ± 0.005	0.183 ± 0.014	0.865 ± 0.004	-0.393 ± 0.015
Miyaura (SM)	Out-Of-Sample	Test 1	R^2	0.724 ± 0.013	0.751 ± 0.005	0.220 ± 0.000	0.367 ± 0.077	-0.048 ± 0.000
	Split	Test 2	R^2	0.816 ± 0.002	0.766 ± 0.004	0.233 ± 0.000	0.589 ± 0.025	-0.356 ± 0.000
	(Catalyst)	Test 3	R^2	0.667 ± 0.002	0.673 ± 0.013	0.096 ± 0.000	0.269 ± 0.046	-0.703 ± 0.000
		Test 4	R^2	0.505 ± 0.007	0.564 ± 0.006	0.030 ± 0.000	0.194 ± 0.035	-0.744 ± 0.000
Buchwald-	Random split	Random (70/30)	RMSE	7.587 ± 0.472	8.568 ± 0.610	11.371 ± 0.557	14.889 ± 0.542	19.118 ± 0.321
Hartwig (BH)	Out-Of-Sample	Test 1	RMSE	8.282 ± 0.549	9.158 ± 0.232	11.579 ± 0.000	15.913 ± 1.154	19.053 ± 0.000
	Split (Reagent)	Test 2	RMSE	7.581 ± 0.163	7.658 ± 0.079	10.017 ± 0.000	15.840 ± 1.693	18.438 ± 0.000
		Test 3	RMSE	12.162 ± 0.271	12.895 ± 0.311	12.237 ± 0.000	20.185 ± 1.072	20.928 ± 0.000
		Test 4	RMSE	11.480 ± 0.146	12.414 ± 0.273	11.133 ± 0.000	24.105 ± 2.314	17.230 ± 0.000
Suzuki-	Random split	Random (70/30)	RMSE	9.755 ± 0.116	10.074 ± 0.158	25.331 ± 0.060	10.275 ± 0.092	33.067 ± 0.243
Miyaura (SM)	Out-Of-Sample	Test 1	RMSE	15.750 ± 0.362	14.985 ± 0.150	26.507 ± 0.000	23.835 ± 1.487	30.721 ± 0.000
	Split	Test 2	RMSE	12.606 ± 0.067	14.227 ± 0.117	25.763 ± 0.000	18.843 ± 0.573	34.251 ± 0.000
	(Catalyst)	Test 3	RMSE	14.440 ± 0.046	14.310 ± 0.294	23.778 ± 0.000	21.380 ± 0.672	32.644 ± 0.000
		Test 4	RMSE	18.647 ± 0.126	17.490 ± 0.122	26.092 ± 0.000	23.786 ± 0.509	34.993 ± 0.000

Results of fine-tuned model learning performance on the downstream test set and the held-out pre-training test set across each epoch on the SM dataset are presented in Fig. S7. The model performance was evaluated using L1-loss throughout the training process. Also, the model prediction performance of the best-epoch model are shown in Table S7.

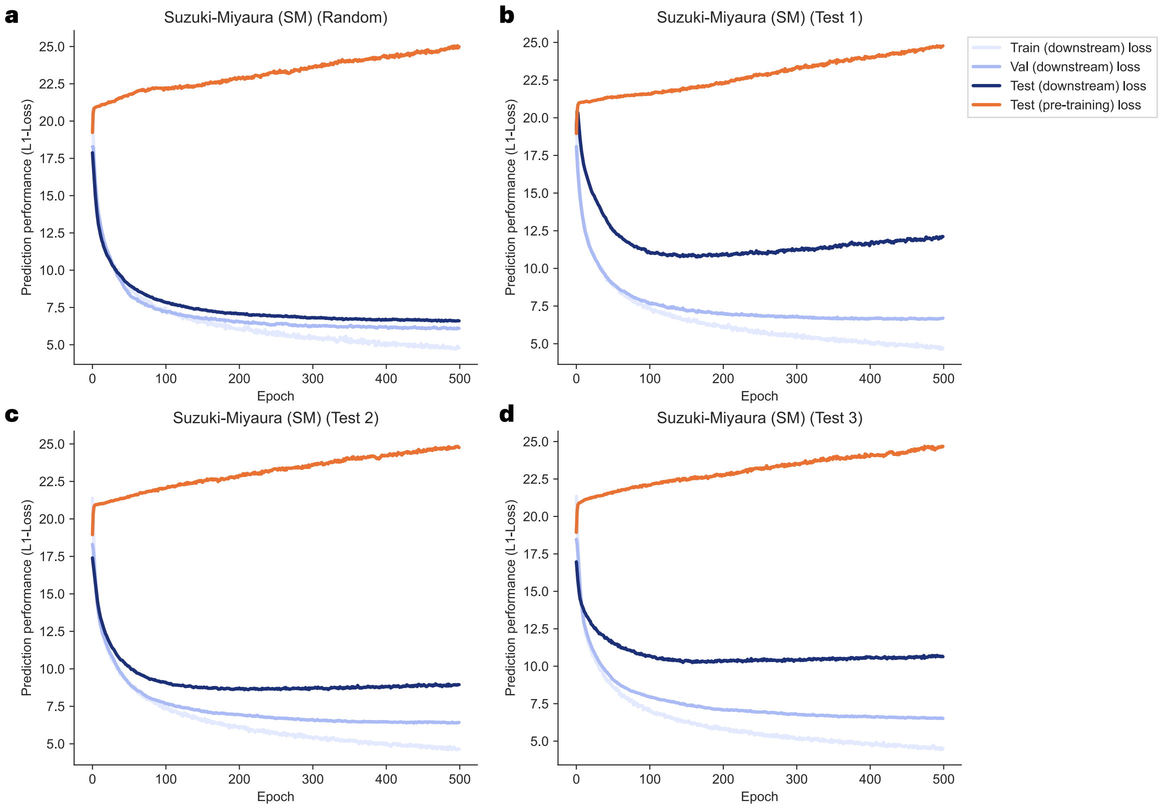

Fig. S7: Model learning performance over fine-turning epoch measured in L1-loss comparing between downstream train, validation, and test set and held-out pretraining test set on SM dataset.

Table S7: Model performance on held-out pre-training test set using different trained model stages

Model	Model performance		
	MAE	RMSE	R2
Model pre-trained on pre-training dataset	17.0572	23.0900	0.379
Model fine-tuned on SM (Random) dataset	24.7436	30.6792	-0.0963
Model fine-tuned on SM (Test 1) dataset	24.2425	29.9675	-0.046
Model fine-tuned on SM (Test 2) dataset	24.2376	30.0517	-0.0519
Model fine-tuned on SM (Test 3) dataset	24.3269	30.1045	-0.0556
Model fine-tuned on SM (Test 4) dataset	24.1149	29.9005	-0.0413

Supplementary Note 6 Latent Space Analysis

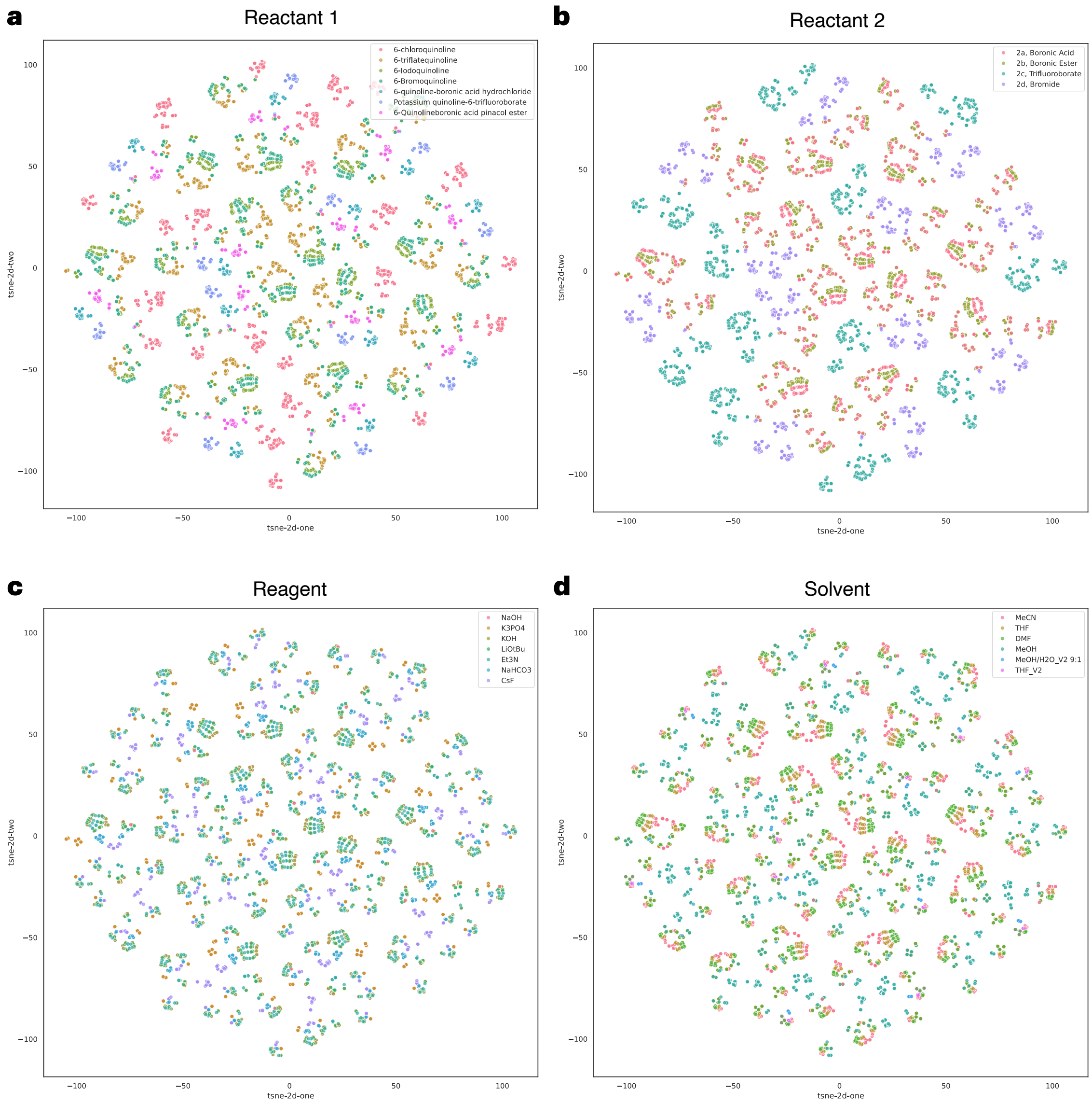

Fig. S8: Embedding of latent space for Suzuki–Miyaura (SM) Test 1 reactions by reaction components. The embedding, visualized using t-SNE, represents all reactions in the Suzuki–Miyaura (SM) Test 1 dataset and is labeled by reactant 1, reactant 2, reagent, and solvent.

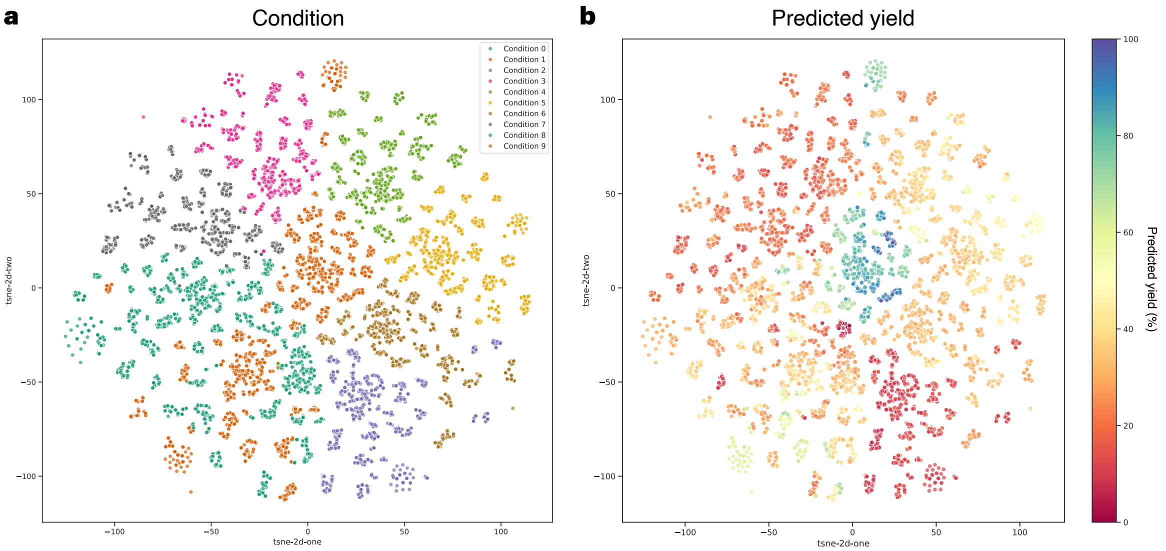

Fig. S9: Embedding of latent space for Suzuki–Miyaura (SM) Test 1 reactions using random set of conditions and latent space. The embedding, visualized using t-SNE, represents all 1,000 random latent space for each condition of the Suzuki–Miyaura (SM) Test 1 dataset.

Supplementary Note 7 Generation Performance

Generation performance for different sampling approaches are displayed in Table S8.

Table S8: Model generation performance

Dataset	Type	Validity (%)	Uniqueness (%)	Novelty (%)	IntDiv	SNN	FCD	Validity (Task) (%)
**SM (Random)**	Random latent + Random condition (no constraint space)	99.95	92.74	92.73	0.88	0.19	22.61	0.21
	Random latent + Random condition	100.00	93.27	93.23	0.81	0.27	18.75	2.66
	Around sample + Random condition	100.00	9.79	9.69	0.68	0.82	2.08	71.33
	Around sample + Sample condition	100.00	9.31	9.21	0.67	0.82	2.03	71.27
	Around sample + Sample condition (w/o post-processing)	82.67	3.91	3.81	0.66	0.90	1.74	65.26
**SM (Test1)**	Random latent + Random condition (no constraint space)	99.98	91.91	91.90	0.88	0.17	23.78	0.26
	Random latent + Random condition	100.00	88.33	88.26	0.80	0.29	19.02	3.59
	Around sample + Random condition	100.00	6.43	6.36	0.63	0.82	2.31	68.14
	Around sample + Sample condition	100.00	5.90	5.83	0.62	0.82	2.26	68.13
	Around sample + Sample condition (w/o post-processing)	84.29	2.96	2.89	0.61	0.88	2.23	64.78
**L-SM**	Random latent + Random condition (no constraint space)	100.00	92.92	92.92	0.85	0.15	29.07	16.61
	Random latent + Random condition	100.00	92.11	92.09	0.86	0.18	21.11	26.67
	Around sample + Random condition	100.00	63.72	63.53	0.89	0.36	10.55	60.53
	Around sample + Sample condition	100.00	62.45	62.26	0.89	0.36	10.45	60.78
	Around sample + Sample condition (w/o post-processing)	34.84	12.46	12.28	0.87	0.57	5.61	27.65
**CC**	Random latent + Random condition (no constraint space)	100.00	98.46	98.46	0.89	0.18	14.21	0.64
	Random latent + Random condition	100.00	99.34	99.34	0.91	0.18	9.88	3.06
	Around sample + Random condition	100.00	80.54	64.91	0.87	0.61	1.49	90.26
	Around sample + Sample condition	100.00	80.16	64.16	0.87	0.62	1.49	91.49
	Around sample + Sample condition (w/o post-processing)	71.46	52.71	36.52	0.85	0.71	1.33	69.16
**PS**	Random latent + Random condition (no constraint space)	99.98	99.64	99.64	0.87	0.13	38.54	6.64
	Random latent + Random condition	99.98	99.65	99.65	0.86	0.14	39.18	3.77
	Around sample + Random condition	100.00	65.05	64.93	0.85	0.51	11.52	69.88
	Around sample + Sample condition	99.99	53.54	53.42	0.84	0.58	9.55	76.95
	Around sample + Sample condition (w/o post-processing)	36.47	6.86	6.75	0.76	0.86	5.53	36.00

Note: Uniqueness means valid and unique molecules. Novelty means valid, unique, and novel molecules from training set. IntDiv means internal diversity. SNN means similarity to a nearest neighbor. FCD means Fréchet ChemNet Distance.

The ablation studies on model generation performance by comparing a model fine-tuned on a pre-trained model with a model trained on the downstream dataset only, using the Asymmetric Pictet–Spengler (PS) dataset, as shown in Fig. S10.

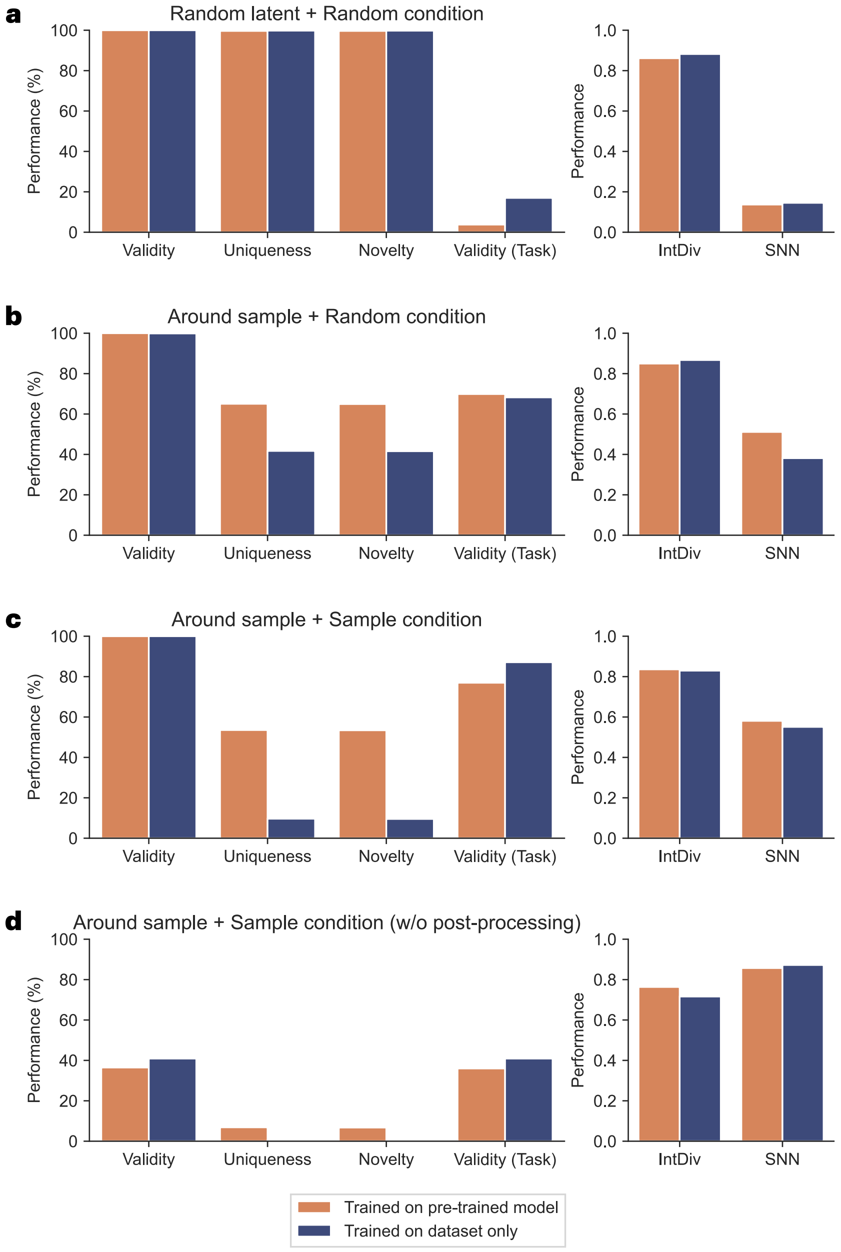

Fig. S10: Model generation performance of ablation study comparing between model trained on pre-trained model and model trained on dataset only on Asymmetric Pictet–Spengler (PS) dataset with different sampling schema.

Supplementary Note 8 Optimization Parameters

The optimization is performed using the Bayesian optimization algorithm from `scikit-optimize` (skopt) [1]. The function `gp_minimize` is used to find optimal solutions. The number of calls is set to 150, with 50 initial points. The Gaussian method is used to model noise. The default acquisition function `gp_hedge` is employed, which probabilistically selects one of the following three acquisition functions (lower confidence bound, negative expected improvement, and negative probability of improvement) at each iteration. All other parameters follow the default configuration provided in the documentation.

Supplementary Note 9 Case Studies

Lewis acid-mediated Suzuki–Miyaura cross-coupling (L-SM).

Model prediction performance of Lewis acid-mediated Suzuki–Miyaura cross-coupling (L-SM) dataset for train and set set in RMSE and R^2 are displayed in Fig. S11 and Fig. S12.

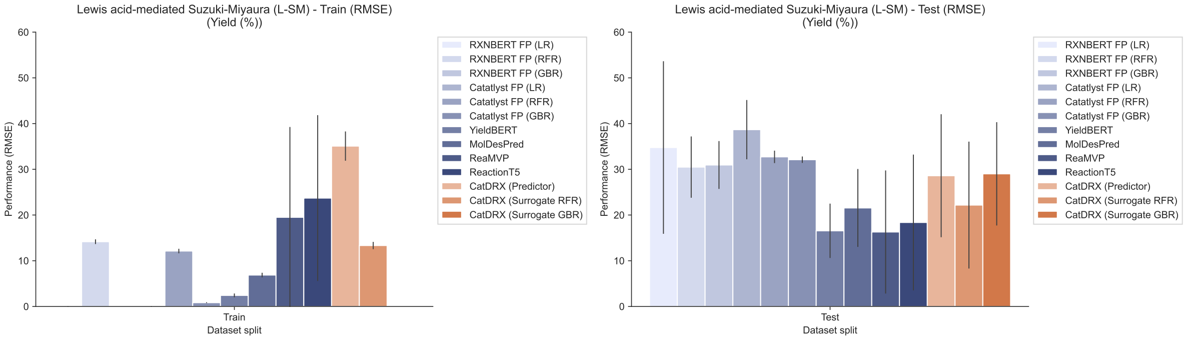

Fig. S11: Model prediction performance of Lewis acid-mediated Suzuki–Miyaura cross-coupling (L-SM) dataset in RMSE

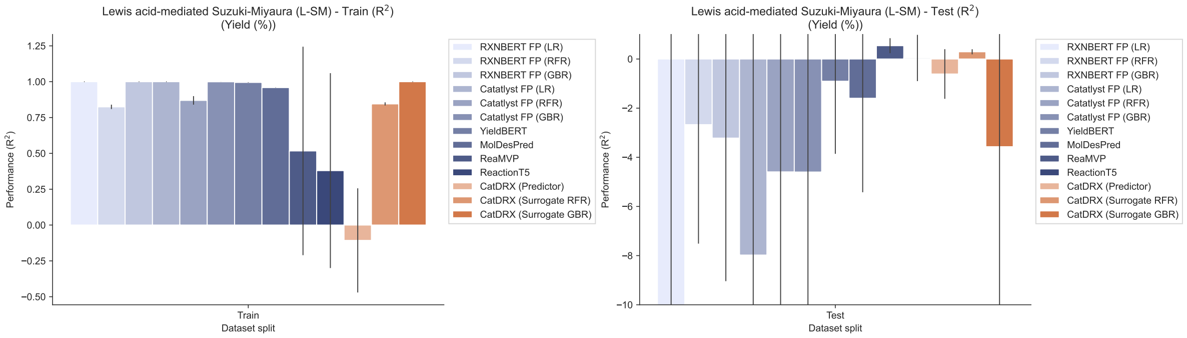

Fig. S12: Model prediction performance of Lewis acid-mediated Suzuki–Miyaura cross-coupling (L-SM) dataset in R^2

List of filtering rules and number of filtered generated molecules from different generation approaches averaged from three different model seeds using mentioned criteria are displayed in Table S9 and Table S10.

Table S9: Filtering rules and optimization conditions for L-SM dataset

Filtering rules	Optimization conditions
Number of fragment is 1	Predicted yield is 100
Contains P atom or N atom	Complies with the filtering rules
Number of neighbors of P is 3	Similarity to training set is not less than 0.25
P is not in 3-membered ring	
Number of neighbors of O does not exceed 2	

Table S10: Number of filtered generated molecules from different generation approaches averaged from three different model seeds

Approach		From generation (around target molecule)	From generation (random in training space)	From optimization
Total generated molecules		10000 (100.00%)	10000 (0.00%)	500 (100.00%)
Total valid and unique molecules		5352 (53.52%)	8943 (89.43%)	342 (68.47%)
Filtering rules	Number of fragment is 1	2716 (27.16%)	2170 (21.70%)	336 (67.20%)
	Contains P atom or N atom	2436 (24.36%)	1844 (18.44%)	336 (67.20%)
	Number of neighbors of P is 3	1251 (12.51%)	571 (5.71%)	331 (66.27%)
	P is not in 3-membered ring	1138 (11.38%)	512 (5.12%)	331 (66.27%)
	Number of neighbors of O does not exceed 2	1091 (10.91%)	466 (4.66%)	331 (66.27%)
Does not exist in training set		1077 (10.77%)	463 (4.63%)	321 (64.13%)
Final candidate molecules		1077 (10.77%)	463 (4.63%)	321 (64.13%)

C-C cross-coupling (CC)

The filtering rules and optimization conditions for Pd-complex optimization are presented in Table S11. A summary table showing the number of candidates remaining at each filtering step is also provided as shown in Table S12. Since the filtering rules are applied during the optimization cycle to promote the generation of promising Pd-complex candidates, most of the generated complexes comply with these rules.

Table S11: Filtering rules and optimization conditions for CC dataset

Filtering rules	Optimization conditions
Number of fragments is 3	Binding energy at -27.55 kcal mol^{-1}
Contains metal atom of Pd	Complies with the filtering rules
Contains P, N, or O atom	Similarity to training set is not less than 0.2
Number of neighbors of P is 3	
Number of neighbors of N does not exceed 3	
Number of neighbors of O does not exceed 2	
Does not contain 3/4-membered ring	

Table S12: Number of filtered generated molecules for Pd-complex optimization

Approach		Optimization for Pd Complexes
Total generated molecules		500 (100.00%)
Total valid and unique molecules		422 (84.40%)
Filtering rules	Number of fragments is 3	282 (56.40%)
	Contains metal atom of Pd	282 (56.40%)
	Contains P, N, or O atom	282 (56.40%)
	Number of neighbors of P is 3	277 (55.40%)
	Number of neighbors of N does not exceed 3	276 (55.20%)
	Number of neighbors of O does not exceed 2	275 (55.00%)
	Does not contain 3/4-membered ring	268 (53.60%)
Does not exist in training set		245 (49.00%)
Final candidate molecules		245 (49.00%)

Benchmark comparing binding energy (kcal mol^{-1}) between predicted values from CatDRX, calculated values from Gaussian16 with the 3-21G basis set, and calculated values from Gaussian16 with the SDD and 6-31G(d,p) basis sets is shown in Table S13. We performed simulations using the same settings in Gaussian16 but with a larger basis set. According to the Gaussian16 documentation, the 6-31G basis set is only applicable to atoms from H to Kr and therefore cannot be used for palladium. Following the approach of [2], we used SDD for the Pd atom and 6-31G(d,p) for the other atoms. As shown, most results align well with previous experiments. Note that, since our model was trained using the original 3-21G setup, some differences may occur between the predicted values and those obtained with the larger basis set for certain complexes.

Table S13: Benchmark comparing binding energy (kcal mol^{-1}) between predicted values from CatDRX, calculated values from Gaussian16 with 3-21G basis set, and calculated values from Gaussian16 with SDD and 6-31(d,p) basis set.

No	Complex	Predicted	DFT Calculated	
			Basis set: 3-21G	Basis set: SDD for Pd atom 6-31G(d,p) for others
1	[Pd].CP(F)F.Cc1ccc(P(c2ccc(C)cc2)c2ccc(C)cc2)cc1	-31.36	-31.74	-25.31
2	[Pd].CC(C)(C)CP(C(C)(C)C)C(C)(C)C.Clc1ccncn1	-26.96	-26.31	-34.66
3	[Pd].Cc1ccccc1P(c1ccccc1C)c1ncccc1C. Fc1ccc(P(c2ccc(F)cc2)c2ccc(F)cc2)cc1	-26.29	-29.44	-25.20
4	[Pd].CP(C)c1cccnc1.FP(F)F	-25.97	-24.00	-23.45
5	[Pd].Cc1ccc(P(c2ccc(C)cc2)c2ccc(F)cc2)cc1.FCCNCCF	-28.99	-31.07	-41.51

Asymmetric Pictet–Spengler (PS)

The filtering rules are displayed in Table S14. There is no optimization process performed for this case study. The summary table showing the number of candidates for each step of filtering for specific reaction conditions is displayed in Table S15.

Table S14: Filtering rules for PS dataset

Filtering rules
Number of fragments is 1

Table S15: Number of filtered generated molecules for generation with specific reaction conditions.

Approach		Generation based on condition		
		con_0	con_1	con_6
Total generated molecules		10000 (100.00%)	10000 (100.00%)	10000 (100.00%)
Total valid and unique molecules		6076 (60.76%)	6063 (60.63%)	7536 (75.36%)
Filtering rules	Number of fragment is 1	3290 (32.90%)	3409 (34.09%)	3880 (38.80%)
Does not exist in training set		3285 (32.85%)	3407 (34.07%)	3880 (38.80%)
Final candidate molecules		3285 (32.85%)	3407 (34.07%)	3880 (38.80%)

Supplementary References

[1] Head, T., MechCoder, Louppe, G., Shcherbatyi, I., fcharras, Vinícius, Z., cmmalone, Schröder, C., nel215, Campos, N., Young, T., Cereda, S., Fan, T., rene-rex, Shi, K.K., Schwabedal, J., carlosdanielcsantos, Hvass-Labs, Pak, M., SoManyUsernamesTaken, Callaway, F., Estève, L., Besson, L., Cherti, M., Pfannschmidt, K., Linzberger, F., Cauet, C., Gut, A., Mueller, A., Fabisch, A.: Scikit-optimize/scikit-optimize: V0.5.2, v0.5.2, Zenodo (2018). https://doi.org/10.5281/zenodo.1207017 . https://doi.org/10.5281/zenodo.1207017

[2] Ma, S., Cao, Y., Shi, Y.-F., Shang, C., He, L., Liu, Z.-P.: Data-driven discovery of active phosphine ligand space for cross-coupling reactions. Chemical science **15**(33), 13359–13368 (2024)
